# Conscious prone positioning during non-invasive ventilation in COVID-19 patients: experience from a single centre

**DOI:** 10.12688/f1000research.25384.1

**Published:** 2020-07-31

**Authors:** Helmi C. Burton-Papp, Alexander I. R. Jackson, Ryan Beecham, Matteo Ferrari, Myra Nasim-Mohi, Michael P. W. Grocott, Robert Chambers, Ahilanandan Dushianthan, University Hospital Southampton Critical Care Team, REACT COVID Investigators

**Affiliations:** 1 General Intensive Care unit, University Hospital Southampton NHS Foundation Trust, Southampton, Hampshire, SO16 6YD, UK; 2 NIHR Southampton Clinical Research Facility and NIHR Southampton Biomedical Research Centre, University Hospital Southampton NHS Foundation Trust, Southampton, Hampshire, SO16 6YD, UK; 3 Faculty of Medicine, University of Southampton, Southampton, Hampshire, SO16 6YD, UK

**Keywords:** COVID-19, Intensive Care, Non-invasive ventilation, Prone

## Abstract

Critically ill patients admitted to hospital following SARS-CoV-2 infection often experience hypoxic respiratory failure and a proportion require invasive mechanical ventilation to maintain adequate oxygenation. The combination of prone positioning and non-invasive ventilation in conscious patients may have a role in improving oxygenation. The purpose of this study was to assess the effect of prone positioning in spontaneously ventilating patients receiving non-invasive ventilation admitted to the intensive care.  Clinical data of 81 patients admitted with COVID 19 pneumonia and acute hypoxic respiratory failure were retrieved from electronic medical records and examined. Patients who had received prone positioning in combination with non-invasive ventilation were identified.

A total of 20 patients received prone positioning in conjunction with non-invasive ventilation. This resulted in improved oxygenation as measured by a change in PaO _2_/FiO _2_ (P/F) ratio of 28.7 mmHg while prone, without significant change in heart rate or respiratory rate. Patients on average underwent 5 cycles with a median duration of 3 hours. There were no reported deaths, 7 of the 20 patients (35%) failed non-invasive ventilation and subsequently required intubation and mechanical ventilation. In our cohort of 20 COVID-19 patients with moderate acute hypoxic respiratory failure, prone positioning with non-invasive ventilation resulted in improved oxygenation. Prone positioning with non-invasive ventilation may be considered as an early therapeutic intervention in COVID-19 patients with moderate acute hypoxic respiratory failure.

## Introduction

The critical illness characterised by the SARS-CoV-2 viral infection (Coronavirus disease 2019; COVID-19) often results in respiratory symptoms leading to acute hypoxic respiratory failure (AHRF) necessitating mechanical ventilation. Supplemental oxygen is the initial mainstay of therapy. Non-invasive ventilation (NIV) has been shown to be a successful alternative to mechanical ventilation in the early stages of the related coronavirus severe acute respiratory syndrome (SARS) and in COVID-19 without a subsequent need for invasive mechanical ventilation ^1, 2^. Despite COVID-19 being a novel single disease entity, various phenotypic variations based on biological markers of immunity, prothrombotic features, ventilator mechanics and radiological changes have been documented ^3^. Consequently, the response to specific therapies may vary between COVID-19 patients with AHRF.

Prone positioning has been often adopted to improve gas exchange in mechanically ventilated patients with moderate to severe refractory hypoxia associated with acute respiratory distress syndrome (ARDS). Prone position was not associated with an improved outcome in a Cochrane review of ARDS patients receiving invasive mechanical ventilation ^4^. However, subgroup analysis demonstrated a better outcome among those patient groups with severe ARDS. The Proning Severe ARDS (PROSEVA) multicentre randomised-controlled trial has demonstrated improved survival in patients with severe ARDS who had received early, long sessions of prone positioning when compared to the control group of supine patients ^5^. Despite most COVID-19 patients with AHRF fulfilling the ARDS clinical definition, there are demonstrable variations in response to therapy between COVID-19 lung disease and ARDS. Although the use of prone position in COVID-19 AHRF has not been formally evaluated by clinical trials, its use is supported by several international guidelines, including The Surviving Sepsis Campaign and ANZICS COVID-19 guidelines ^6, 7^. Moreover, recently a study demonstrated improved oxygenation during a single episode of prone positioning in awake non-ventilated COVID-19 patients ^8^, while another has demonstrated its feasibility in a non-critical care environment ^9^. A recent review of conscious proning in ARDS and COVID-19 infection which included a handful of studies with small number of patients and concluded short term improvements in the oxygenation ^10^. Here we build on this literature offering an examination of changes in oxygenation, as measured by PaO _2_/FiO _2_, across multiple episodes of prone positioning in conscious patients, with moderate to severe hypoxia, undergoing non-invasive ventilation following admission to the intensive care unit for advanced respiratory support.

## Methods

### Study background

We collected data from all COVID-19 reverse transcriptase polymerase chain reaction (RT-PCR)-confirmed (nasal and throat swab specimens) admissions to the General Intensive Care Unit, University Hospital Southampton NHS Foundation Trust between 4 ^th^ March 2020 and 11 ^th^ May 2020. When clinical stability allowed, all patients presenting with AHRF needing additional respiratory support, beyond standard oxygen therapy, were trialled on non-invasive ventilation (either continuous positive airway pressure (CPAP) or bilevel positive airway pressure ventilation (BiPAP)). Moreover, depending on their tolerability, patients were encouraged to self-prone. All patients that deteriorated while on NIV went on to have endotracheal intubation and mechanical ventilation.

### Ethical considerations

Ethical approval was obtained as part of the REACT COVID observational study (A longitudinal Cohort Study to facilitate Better understanding and Management of SARS-CoV-2 infection from hospital admission to discharge-across all levels of care): REC Reference 17/NW/0632 SRB Reference Number; SRB0025. Due to the nature of the study, the need for individual informed consent was waived. 

### Data collection and processing

Data was collected from our electronic clinical information (CIS) system using a combination of semi-automated data extraction and manual collection. We collected baseline demographics (age, gender, duration of symptoms, critical illness severity scores, presence of other comorbidities and laboratory variables), medical treatments received, ventilatory parameters, and position data. Position data are recorded hourly by the ICU nursing staff in the CIS and from this we derived number of prone cycles, timing and total duration. We defined NIV failure as the need for mechanical ventilation. On admission we also collected severity indices such as Acute Physiology and Chronic Health Evaluation (APACHE II), Sequential Organ Failure Assessment (SOFA) scores and oxygenation status. Arterial blood gas (ABG) results were collected alongside patient observations (heart rate (HR) and respiratory rate (RR)) from our CIS to measure change in parameters before, during and after prone position. We assessed the change in oxygenation by measuring the change in the arterial oxygen partial pressure (PaO _2_ in mmHg) to fractional inspired oxygen (FiO _2_) ratio (ΔP/F), change in respiratory rate (ΔRR) and heart rate (ΔHR).

### Statistical analysis

All statistical analysis and data processing were performed using R (R Core Team, Vienna, Austria). Data were tested for normality; those found to be normally distributed were presented as mean and standard deviations (SDs), while non-normally distributed data are presented as median and IQR. Changes in physiological parameters across prone cycles were tested using a paired t-test and are presented and mean and 95% confidence interval. Significance testing between groups was carried out using an independent 2-group t-test for normally distributed variables and the Mann-Whitney U-test for non-normally distributed variables.

## Results

There were 81 COVID-19-confirmed patients admitted to the General Intensive Care Unit between 4 ^th^ of March and 11 ^th^ of May 2020. The outcomes are up to date as of 26 June 2020. Of those, 20 patients (25%) had a combination of both non-invasive ventilation and self-prone positioning. The mean age of these patients was 53.4 ± 8.3, 55% were male and median admission APACHE II and SOFA severity indices were 11.5 (IQR 5) and 3 (IQR 0) respectively. Among those, 7 patients failed NIV and were subsequently intubated (NIV+IMV group). The characteristics of these patients with severity indices are presented in Table 1. Raw results for each patient are available as *Underlying data* ^11^.

**Table 1.** Patient demographics for all patients admitted and received non-invasive ventilation and self-prone positioning.

Demographics	All NIV and prone patients (N=20)	Only NIV (N=13)	NIV and IMV (N=7)
Age, year *	53.4 ± 8.3	54.6 ± 9.1	51.3 ± 6.8
Male sex, n (%)	(55%)	(42.9%)	(61.5%)
Duration of symptoms (days) †	7 (6)	7 (4.25)	9 (6.5)
Admission APACHE II score †	11.5 (5)	11 (4)	11 (10.5)
Admission SOFA Score †	3 (0)	3 (1)	4 (3)
Admission PaO _2_/FiO _2_ (mmHg) *	123 ± 27.8	127 ± 26.4	116 ± 31.0
Co-morbidities, n (%)			
BMI ≥30 (kg/m ^2^)	11 (55)	6 (46)	5 (71)
Diabetes mellitus	3 (15)	2 (15)	1 (14)
Chronic respiratory illness	7 (35)	4 (20)	2 (29)
Ischaemic Heart Disease	1 (5)	1 (8)	0
Congestive Cardiac Failure	1 (5)	0	1 (14)
Immunosuppression	2 (10)	0	2 (29)
Admission bloods †			
Urea (mmol/l)	5.6 (3.8)	4.8 (4)	6.3 (6.6)
Creatinine (μmol/l)	68 (35)	68 (22)	65 (60)
eGFR	90 (20.5)	90 (20)	90 (32.5)
Bilirubin (μmol/l)	11.5 (4)	12 (4)	11 (4)
WBC (n x 10 ^9^/l)	9.4 (6.1)	11 (5.9)	7.9 (6.5)
Lymphocytes (n x 10 ^9^/l)	0.95 (0.5)	1 (0.4)	0.8 (0.6)
CRP (mg/l)	127 (105)	121 (92)	133 (107)
INR	1.2 (0.13)	1.2 (0.1)	1.2 (0.15)
Ferritin (ng/ml)	1491 (1583)	2179 (1625)	872 (427)
HS Troponin I (ng/l)	10 (11.5)	9.5 (5)	15 (21.5)
Lactate dehydrogenase (U/l)	1021 (463)	1202 (574)	900 (224)
D-Dimer (μg/l)	545 (676)	494 (766)	942 (521)
Creatine Kinase (U/l)	150 (197)	184 (223)	129 (54)
Medications given			
Antibiotics, n (%)	20 (100)	13 (100)	7 (100)
Antivirals, n (%)	8 (40)	4 (31)	4 (57)
Corticosteroids, n (%)	5 (25)	2 (15)	3 (43)
Other immunosuppressive medications, n (%)	2 (10)	0 (%)	2 (29)
Non-Invasive ventilation variables			
CPAP only, n (%)	4 (20)	4 (31)	0 (0)
CPAP and Bilevel, n (%)	16 (80)	9 (69)	7 (100)
Bilevel only, n (%)	0 (0)	0 (0)	0 (0)
% Time on Bilevel Mode, hours †	32.9 (1.8-69.8)	7.1 (0-51.0)	61.4 (29.5-80.1)
CPAP cmH _2_O †	10 (8-10)	10 (8-10)	10 (8.5-10)
IPAP cmH _2_O †	15 (14-16)	15 (14.5-17)	14 (13-15)
EPAP cmH _2_O †	10 (10-10)	10 (10-10)	10 (9-10)
Duration of NIV, hours †	82.4 (53.7-134.8)	105 (56.3-154)	57 (52.4-99.4)

*Data are presented in mean ± standard deviation. †Data are presented in median and interquartile range. APACHE II, Acute Physiology and Chronic Health Evaluation II score; BMI, body mass index; CPAP, continuous positive airway pressure; CRP, C-reactive protein; eGFR, estimated glomerular filtration rate; EPAP, expiratory positive airway pressure; HS Troponin, High Sensitivity Troponin; INR, International Normalised Ratio; IPAP, inspiratory positive airway pressure; PaO _2_/FiO _2_, ratio of arterial oxygen partial pressure to fractional inspired oxygen; SOFA, Sequential Organ Failure Assessment score; WBC, white blood cell count.

**Figure 1.**
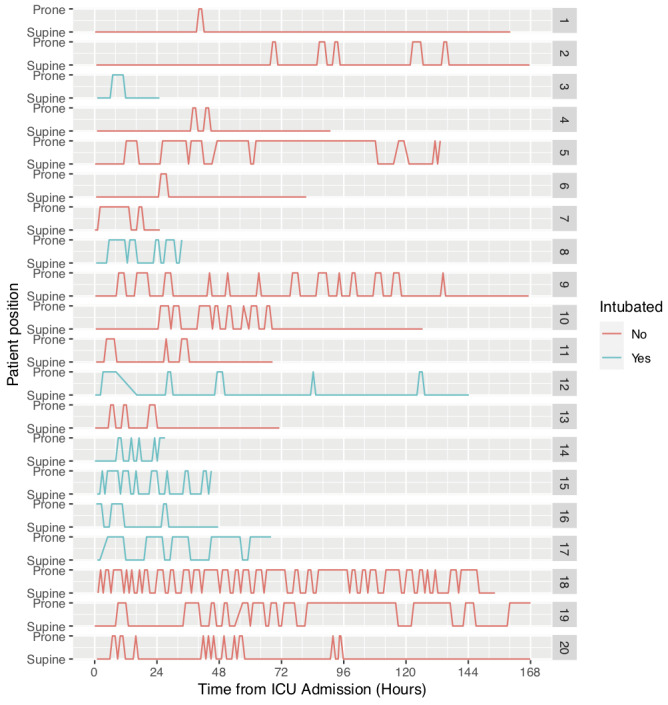
Time spent prone vs time spent supine (hours) for each patient throughout their admission. Patients that failed non-invasive ventilation and required invasive mechanical ventilation (NIV+IMV group) are shown in blue and non-invasive ventilation (NIV) only group in red.

There was a total number of 141 prone cycles performed between these patients. Although the duration of each cycle was variable between cycles and among individual patients, the median duration for each cycle was 3 hours (IQR 2) and the number of proning cycles per patient was 5 (IQR 6.3). Five patients continued with prone positioning beyond 96 hours. Most cycles (46%), were between 1 and 3 hours, a summary detailing prone cycles and duration characteristics is shown in Table 2. Additionally, Figure 1 is a graphical representation of each patient’s time on NIV, demonstrating time spent in prone and supine positions. It follows each of the 20 patients, tracking their position throughout the duration of their admission, up until discharge from ICU or point of intubation. 

**Table 2.** Prone cycle characteristics for all patients.

Prone characteristics	All NIV and prone patients (N =20)	Only NIV (N=13)	NIV and IMV (N=7)
Duration of Cycle (minutes)	Number of cycles		
• <60	32	23	9
• 61–180	69	52	17
• 181–360	22	18	4
• >360	18	11	7
Total number of cycles, n	141	108	33
Cycles per patient, n *	5 (6.25)	5 (9)	5 (3)
Duration of each cycle, hour *	3 (2)	3 (2)	3 (3)
Percentage time prone (%) *	18.3 (31)	11.8 (27)	23.6 (23.5)

*Median (Interquartile range). IMV, Invasive mechanical ventilation; NIV, non-invasive mechanical ventilation.

**Figure 2.**
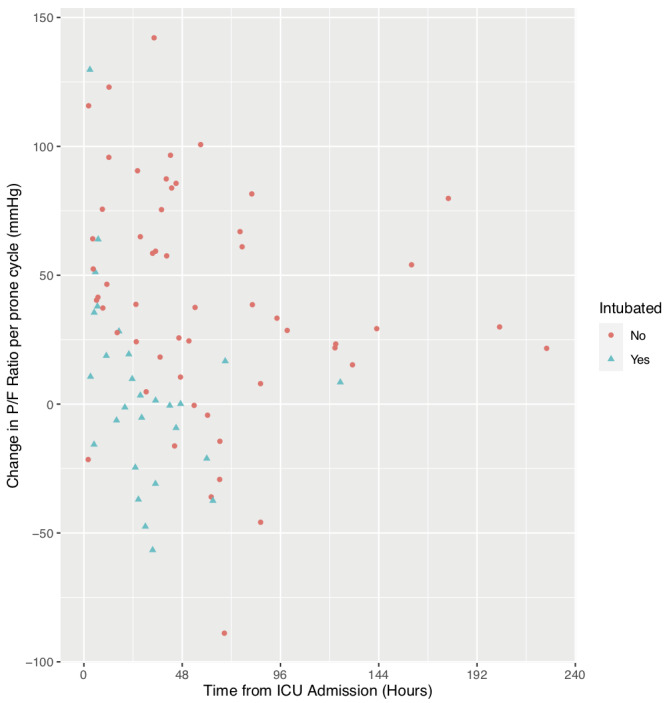
Pictorial representation of changes in the PaO2/FiO2 ratio for each prone cycle from all patients. Patients that failed non-invasive ventilation and required invasive mechanical ventilation (NIV+IMV group) are shown as blue dots and non-invasive ventilation (NIV) only group in red dots.

The outcomes are shown in Table 3. Overall, there was an increment in P/F ratio of 28.7 mmHg (3.83 kPa) (95% CI 18.7-38.6 mmHg, p<0.01) with no change in the heart rate or respiratory rate. The patients who had a successful NIV and prone trial (65%) had a greater increment in P/F ratio of 40.8 mmHg (5.44 kPa) (95% CI 28.8-52.7 mmHg, p<0.01), while those who went on to be intubated did not have a significant improvement in P/F ratio (+5.06 mmHg (0.67 kPa), 95% CI -9.5-19.7 mmHg, p=0.48). Those patients who avoided IMV had a significantly shorter length of hospital stay (11 vs. 28 days, p<0.01) but no other significant differences in outcome or baseline characteristics were observed. Most of the improvement was seen within 24 hours and after this time point the incremental beneficial effect is less for both groups ( Figure 2). On average the NIV+IMV group spent 24% of their intensive care unit time pre-intubation in the prone position compared to only 12% for the NIV only group.

**Table 3.** Outcome variables.

Outcomes	All NIV and prone patients (N =20)	Only NIV (N=13)	NIV and IMV (N=7)
Δ PaO _2_/FiO _2_ (mmHg) *‡	+ 28.7 (95%CI 18.7-38.6)	+ 40.8 (95%CI 28.8-52.7)	+ 5.06 (95%CI -9.5-19.75)
Δ Respiratory rate (per min) *	-0.98 (95%CI -2.0-0.04)	-1.27 (95%CI -2.4- -0.1)	-0.09±6.45 (95%CI -2.3-2.1)
Δ Heart rate (per min) *	-1.08±7.69 (95%CI -17.9-1.7)	-1.24 (95%CI -2.6-0.17)	-0.61 (95%CI -3.8-2.5)
Response to prone positioning, n			
• Mean Δ PaO _2_/FiO _2_ < 0 mmHg	3 (15%)	2 (15.9%)	1 (14.3%)
• Mean Δ PaO _2_/FiO _2_ 0-7.5 mmHg	4 (20%)	0 (0.0%)	4 (57.1%)
• Mean Δ PaO _2_/FiO _2_ > 7.5 mmHg	13 (65%)	11 (84.6%)	2 (28.5%)
Length of ICU stay (days) †§	6 (7.3)	5 (5.0)	14 (17.0)
Length of hospital stay (days) †§	17 (16.3)	11 (9)	28 (5.5)
Discharged home, n (%)	20 (100%)	13 (100%)	7 (100%)
Transfer to SARF centre, n (%)	2 (10%)	0 (%)	2 (29%)
Death, n (%)	0 (%)	0 (%)	0 (%)

*Mean (95% CI). †Median and (Interquartile range). ‡P value <0.05 using Welch’s t-test; §P value <0.05 using Mann-Whitney U-Test. CI, confidence interval; ICU; intensive care unit; IMV, invasive mechanical ventilation; NIV, non-invasive ventilation; PaO _2_/FiO _2_, ratio of arterial oxygen partial pressure to fractional inspired oxygen; SARF, severe acute respiratory failure.

There were no deaths recorded in either group and all patients who had successful NIV and prone positioning without the need for mechanical ventilation were discharged home. Among those 7 patients who failed NIV (35%), 2 were transferred to the regional extra-corporeal membranous oxygenation (ECMO) centre, both were later discharged home ( Table 2). There were no reported cases of any adverse events from these proning episodes. 

## Discussion

This is a single-centre retrospective observational study of the clinical outcomes of COVID-19 patients who received a combination of conscious proning and non-invasive ventilation as part of their initial ventilation strategy. Conscious proning and non-invasive ventilation was found to be feasible in 20 patients. Despite variations in patient tolerability and cycle duration, oxygenation improved during prone position period without adverse changes in respiratory rate or heart rate. This response was most marked in patients that did not require escalation to invasive ventilation (65%). Patients that went on to require invasive mechanical ventilation (35%) did not have an improvement in their P/F ratio: modest improvements observed in the first 24 hours ( Figure 2) were not sustained beyond this initial period. These patients were similar to patients who received only NIV with respect to APACHE II, SOFA scores and degree of hypoxia defined by P/F ratio on admission.

The strain that the coronavirus pandemic has placed on national healthcare infrastructure is unprecedented. Although there is only limited data available on the effectiveness of non-invasive ventilation (NIV) in COVID-19, early provision of NIV in moderate to severe acute hypoxic respiratory failure is associated with reduced ICU mortality and intubation rate ^12^. NIV is less resource intensive than IMV and can be managed outside of a critical care environment. NHS England COVID-19 specific guidance suggests that prone position may be of use in NIV patients to improve ventilation/perfusion mismatch, work of breathing and oxygenation ^13^. Whilst the use of prone positioning is well defined and has been extensively evaluated in patients with ARDS by several randomised controlled trials, the benefits of prone positioning in awake, conscious patients with moderate to severe AHRF or ARDS has not been fully explored. Reports from the COVID-19 pandemic from various countries suggests prone positioning in spontaneously ventilating patients may be of value in preventing progression to mechanical ventilation.

Our findings suggest that for a proportion of COVID-19 patients with moderate AHRF (P/F ratio <200 mmHg), non-invasive ventilation in combination with conscious proning can lead to an improvement in oxygenation, less requirement for invasive ventilation and potentially better overall outcomes. All patients with a sustained (>24 hours) positive response to prone positioning avoided intubation, affording them a shorter overall length of hospital stay. However, 35% of patients receiving NIV progressed to invasive mechanical ventilation, despite a similar overall number and duration of proning cycles per patient between both groups. Although none of these patients died, they required a much longer period of hospitalisation and therefore caution is advised when implementing non-invasive ventilation and prone positioning outside of a critical care environment with adequate resources to manage NIV failure.

The use of prone positioning in COVID-19 pneumonia is supported by our understanding of the pathophysiology of the disease. There is inhomogeneity in the lungs, and CT scans of COVID-19 patients typically shows areas of peripheral ground glass changes that later develop into linear consolidations ^14^. Areas of exudation, macrophage infiltration, fibrosis and mucous plugs are typical findings on autopsy of deceased patients with COVID 19 ^15^. Placing patients in the prone position may help to drain secretions from the lung peripheries, improve lung dyshomogeneity, recruitment and ventilation/perfusion mismatch. Many international guidelines recommend prone positioning for intubated and mechanically ventilated patients for these reasons, so it seems reasonable to conclude that similar benefits may be gained by prone positioning in non-invasively ventilated patients with similar underlying pathology.

There are obvious risks associated with treating patients with AHRF with non-invasive ventilation. The primary risk to the patient is inappropriate delay in intubation and ventilation. COVID-19 AHRF frequently meets the criteria for ARDS and previous studies have suggested that in ARDS, delaying intubation due to the use of NIV is associated with increased mortality ^16^. Likewise, the SARS-CoV-2 virus is transmitted by respiratory droplets; non-invasive ventilation has been shown to produce droplets of >10 μm in size that are largely deposited on surfaces within a 1 meter radius ^17^. Therefore, NIV could potentially increase the risk of virus transmission to individuals in close proximity with the patient. This needs to be a consideration when devising guidelines for personal protective equipment and appropriate cohort allocation of infected patients to minimise infection risk.

There are some limitations of this study. This is a single-centre, retrospective cohort study only limited to small number of COVID-19 patients admitted to the intensive care unit setting with the option of subsequent escalation of care to mechanical ventilation. Most patients were in general young, with a median age of <60 years old and able to self-prone with non-invasive ventilation. These findings may not be transferable to older patient’s group or patients with severe acute hypoxic respiratory failure with P/F ratio of <100mmHg. The study was observational, there were no set criteria for NIV proning and NIV failure with subsequent endotracheal intubation. The clinical judgement and subsequent interventions provided may have been variable between individual senior clinicians and may not be reflective of other centres. Additionally, position data are only recorded hourly and as such some granularity of the data may be lost when the cycles were much shorter period of less than an hour.

Despite these limitations, our results are in line with the other recently published studies of conscious prone positioning in COVID-19 pneumonia. Several case reports and small (up to 25 patients) observational studies conducted in multiple settings (outside ICU, emergency department) with variations in respiratory support (non-invasive ventilation/high-flow nasal oxygen/standard face mask oxygen therapy) and varying severity of hypoxemia has demonstrated beneficial effects of prone positioning in COVID-19 pneumonia. All these studies suggest conscious prone position is associated with an increment in oxygenation and recovery without the need for mechanical ventilation in most cases ^8– 10^. Our results demonstrate, specifically that a sustained response across multiple cycles, for a period >24 hours, is associated with successful treatment with NIV. Taken together, these results indicate that prone positioning in awake, non-intubated patients, in combination with non-invasive ventilation is feasible and may be considered as an early intervention in COVID-19 respiratory failure, particularly in the context of a severe pandemic to prevent mechanical ventilation and its subsequent complications. They also suggest that a loss of response to prone position may potentially be a sign of NIV failure and warrant early evaluation and consideration for endotracheal intubation.

## Conclusion

In conclusion we demonstrate that prone positioning in conjunction with NIV can improve oxygenation in patients with COVID-19. This can be achieved without significant adverse effects and particularly in those with a sustained response, may avoid intubation. When used in a suitably monitored environment, with access to experienced clinicians able to facilitate invasive mechanical ventilation if required, prone positioning alongside NIV may be a useful tool in treating COVID-19 patients with moderate acute hypoxic respiratory failure.

## Data availability

### Underlying data

Figshare: Conscious proning _Burton-Papp 2020.numbers. https://doi.org/10.6084/m9.figshare.12676565.v2 ^11^.

This project contains de-identified data for each patient, including details of proning and type of non-invasive ventilation received.

Data are available under the terms of the Creative Commons Attribution 4.0 International license (CC-BY 4.0).
