# Supplementary material for: Conscious prone positioning during non-invasive ventilation in COVID-19 patients: experience from a single centre
Source: F1000Res. 2020 Jul 31;9:859. [Version 1] doi: 10.12688/f1000research.25384.1 (PMC7578762; doi:10.12688/f1000research.25384.1)
Supplement: Peer review history

# Peer review: Reviewer response for version 1

**DOI:** 10.5256/f1000research.28008.r72632

**Published:** 2020-10-21

**Authors:** Ivan Pavlov

**Affiliations:** 1 Department of Emergency Medicine, Hôpital de Verdun, Montreal, QC, Canada

This is a retrospective report on a subgroup of 20 patients with severe COVID-19 who were able to self-prone while receiving non-invasive ventilation in the ICU, among a grand total of 81 patients admitted to the ICU during the study period. 

 The primary outcome reported by the authors is change in PaO2/FiO2 ratio while in prone position. Clinical outcomes, such as mortality and the incidence of intubation, are also reported. 

 The retrospective nature of the report precludes any causal inferences with regards to the efficacy of awake proning. However, the authors do show that intubation can indeed be avoided in some patients with moderate ARDS due to COVID-19, and that awake proning is associated with improvements in PaO2/FiO2 ratios. 

 The authors assert that improvement in PaO2/FiO2 ratios predicted avoidance of intubation. Their data do not allow us to conclude whether this improvement is solely the response to awake proning, or natural evolution of patients who were already getting better in the first place, and would have avoided intubation whether or not they were subjected to awake proning. One may also wonder if reverse causality was also at play, and if patients who didn't improve their PaO2/FiO2 ratio were intubated precisely for that reason. Intubation is a soft outcome that relies on treating physician judgement, and it is difficult to disentangle cause and effect in this scenario. 

 In summary, the authors have shown that PaO2/FiO2 ratios may improve in response to awake proning, and they did not identify any obvious safety signals in this cohort of patients that were able to tolerate awake self-proning. Although this paper does not demonstrate efficacy of awake proning, it does add to the growing body of knowledge on this subject and allows comparisons with other cohorts which used different strategies in terms of duration of proning, as well as the type of respiratory support. Publication of such prelimary data is important, especially while awaiting definitive answers from randomized controlled trials. 

Is the work clearly and accurately presented and does it cite the current literature?

Yes

If applicable, is the statistical analysis and its interpretation appropriate?

Yes

Are all the source data underlying the results available to ensure full reproducibility?

Yes

Is the study design appropriate and is the work technically sound?

Yes

Are the conclusions drawn adequately supported by the results?

Yes

Are sufficient details of methods and analysis provided to allow replication by others?

Yes

Reviewer Expertise:

Emergency medicine; respiratory support with HFNC, awake proning.

I confirm that I have read this submission and believe that I have an appropriate level of expertise to confirm that it is of an acceptable scientific standard.

# Peer review: Reviewer response for version 1

**DOI:** 10.5256/f1000research.28008.r68510

**Published:** 2020-09-04

**Authors:** John Whittle

**Affiliations:** 1 Department of Anesthesiology and Critical Care, Duke University, Durham, NC, USA

In this retrospective observational cohort study, 20 spontaneously ventilating patients admitted to critical care with hypoxic respiratory failure consequent to COVID 19 pneumonia, who underwent proning while receiving non-invasive ventilation were identified from the EHR. The primary outcome examined was a change in PaO2/FiO2 (P/F) ratio while prone as compared with supine, which the authors report as improved while prone.

 In terms of data quality and study design, since this is a retrospective study, no single protocol for awake proning was used, creating a large amount of variability in duration, cycle length and frequency of intervention. Similarly, total length of time spent on NIV, and total number of hours /days spent awake proning appears very varied. This reduces the utility of the data significantly. No comparative statistics are presented between groups (correctly).  

 In terms of trajectory of illness, it would be helpful to plot the overall P/F ratio over time for both intubated and non intubated groups, since from the data available it would appear that those patients who were ultimately intubated started with worse oxygenation and benefited least from the intervention.

 It may also be useful to examine a control group of patients who did not self-prone, but did receive non-invasive ventilation during the study period.

 The data presented do however, provide reassurance in terms of safety of the intervention in terms of hemodynamic and respiratory stability.

 I do not agree that the data presented in this study demonstrate the potential for better overall outcomes with awake proning in COVID-19. More data is required to demonstrate that the trajectory of illness was altered by proning, which is not available in this study. Rather, the observation that patients who avoided intubation overall spent lower percentage of time prone and had a increased response to proning in terms of P/F ratio, suggests that those patients on the trajectory (who are not well described in this study) towards intubation did not have their outcome altered by proning. Rightly, in the discussion the authors discuss the risks associated with applying awake proning.

 In summary Burton-Papp *et al.* have shown that P/F ratio may improve in proned patients on non-invasive ventilation with COVID-19 respiratory failure. Their data also suggests that awake proning on NIV in COVID-19 positive patients is safe in terms of cardiorespiratory stability in those patients who are able to tolerate the intervention. This intervention may therefore provide some clinical benefit, but it is not possible to determine from this study with limited numbers where no control group was presented. What they have not demonstrated is whether proning on NIV alters clinical course, prevents intubation, what protocol is most effective or who might benefit most from this intervention all of which would require prospective study or matched controlled. They further did not describe which features prevented patients from tolerating awake proning on NIV which may be useful in understanding the impact of the intervention.

Is the work clearly and accurately presented and does it cite the current literature?

Yes

If applicable, is the statistical analysis and its interpretation appropriate?

Yes

Are all the source data underlying the results available to ensure full reproducibility?

Yes

Is the study design appropriate and is the work technically sound?

Partly

Are the conclusions drawn adequately supported by the results?

Partly

Are sufficient details of methods and analysis provided to allow replication by others?

Yes

Reviewer Expertise:

Critical Care and Perioperative Medicine

I confirm that I have read this submission and believe that I have an appropriate level of expertise to confirm that it is of an acceptable scientific standard.
